# Timing of food pieces introduction and neurodevelopment: findings from a nationwide birth cohort

**DOI:** 10.1186/s12966-024-01669-5

**Published:** 2024-10-16

**Authors:** Maria Somaraki, Blandine de Lauzon-Guillain, Aurore Camier, Jonathan Y. Bernard, Muriel Tafflet, Marie-Noëlle Dufourg, Marie-Aline Charles, Claire Chabanet, Carole Tournier, Sophie Nicklaus

**Affiliations:** 1 Centre Des Sciences Du Goût Et de L’ Alimentation, CNRS, INRAE, Institut Agro, Université Bourgogne, 17 Rue Sully, Dijon Cedex, 21065 France; 2 Centre for Research in Epidemiology and Statistics (CRESS), Université de Paris Cité and Université Sorbonne Paris Nord, Inserm, INRAE, Paris, 75004 France; 3 Unité Mixte Inserm-Ined-EFS Elfe, Ined, Aubervilliers Cedex, 93322 France; 4 INRAE, PROBE research infrastructure, ChemoSens facility, 17 Rue Sully, Dijon Cedex, 21065 France; 5 MoISA, University Montpellier, CIRAD, CIHEAM-IAMM, INRAE, Institut Agro, IRD, Montpellier, France

**Keywords:** Motor skills, Language, Cognition, Neurodevelopmental scores, Complementary feeding, Food texture

## Abstract

**Background:**

While complementary feeding can be challenging, little emphasis has been placed on the introduction to food texture/pieces, especially in terms of neurodevelopmental outcomes. This study aims to determine the association between the timing of introduction to food pieces during infancy and neurodevelopment in early childhood. We hypothesized that late introduction to food texture/pieces relates to unfavorable neurodevelopmental outcomes.

**Methods:**

Families (*n* = 18329) were recruited from the general population during the nationwide ELFE (Étude Longitudinale Française depuis l’Enfance) birth cohort in France, and 8511 were selected for a complete case analysis. Age at introduction to food pieces was determined based on repeated assessments during the first year. A range of neurodevelopmental outcomes among children were assessed using validated instruments, i.e. composite scores at 1 and 3.5 years, and a score for language acquisition at 2 years. Risk for developmental delay at 3.5 years was defined based on a developmental quotient (DQ) below 90 according to the child’s chronological age and the respective composite score at this age. We used linear regression modelling to evaluate associations between age at introduction to food pieces and the standardised neurodevelopmental scores, while logistic regression models were used in the analyses according to the risk for developmental delay.

**Results:**

Our findings highlight consistent associations between late introduction to food pieces (i.e., after 10 months, compared to early (before 8 months)) and lower estimates of standardised neurodevelopmental scores at ages 1, 2 and 3.5 years (-0.35 [-0.40; -0.30], -0.15 [-0.20; -0.10] and − 0.18 [-0.23; -0.13], respectively). Infants introduced to pieces late were also more likely to be at risk for developmental delay according to DQ < 90 (OR [95%CI] = 1.62 [1.36; 1.94]).

**Conclusions:**

This study shows that late introduction to food pieces (> 10 months) is related to lower neurodevelopmental scores. Given the challenges that complementary feeding may pose, concerted efforts are required to enhance our understanding of the sensory aspects of early diets and to ultimately provide guidance.

**Supplementary Information:**

The online version contains supplementary material available at 10.1186/s12966-024-01669-5.

## Background

While the recommended timing of complementary feeding, i.e. the transition between milk feeding to family foods when diverse foods are introduced [1], has been well addressed in the literature [1–7], the practices of introduction to food texture/pieces and their implications for child development have been largely understudied. The few studies addressing introduction to nonsmooth foods have underscored its relevance for the development of food preferences [8–11]. Seminal work in the UK showed that, compared to the introduction of lumpy solids to children between 6 and 9 months old, their introduction to children older than 10 months was related to a narrower food repertoire (in terms of fruits, vegetables and legumes) within the first 2 years of life [8], with lasting associations up to age 7 years [9]. Moreover, toddlers at 12 months demonstrated significant variability in their intake of chopped carrots according to familiarity and previous experience with textured foods [10], corroborating earlier observations among infants [12]. These observations align with the general recommendations by the Nutrition Committee of the European Society for Paediatric Gastroenterology, Hepatology, and Nutrition (ESPGHAN) by Fewtrell et al. (2017), which discourage delaying the introduction of textured foods beyond age 10 months [1].

Food texture introduction is an important element of complementary feeding. Relevant recommendations consider it according to developmental readiness, i.e., in relation to certain signs that the child can safely handle textured foods [1, 6]. Such signs are linked to motor skills development, for instance, sitting without support, attempts to self-feed, capacity to hold foods and oral motor and feeding skills [1, 4, 6, 13, 14]. In typically developing infants, readiness for foods other than gruels or purees can manifest as early as 6 months old [6, 11]. In any case, it is recommended that by the age of 8 months, infants have established the consumption of at least minimally textured foods by the provision of food with lumps in it [1, 15]. At this stage, signs of developmental readiness, such as chewing motions [6, 11, 16], are further reinforced by offering increasingly complex textures in food [13, 15, 17–19]. Thus, delayed exposure and introduction to textured foods may discourage achieving the full potential in early childhood development.

Timely exposure to increasingly complex food textures is defined by parental responsiveness to the child’s signs of developmental readiness. In fact, the literature suggests that a critical window for exposure to food pieces does exist, which is defined as the introduction of lumpy foods by 8 to 10 months [1] corresponding to the child’s signs of readiness. Children need to handle increasingly complex textures when they are first ready to do so; otherwise, it is a missed opportunity that entails more challenging feeding later on [9, 12, 20–22]. The ensuing challenges may be a consequence of the lack of familiarity reinforcing food aversion and pickiness [10, 12, 18, 23], the lack of development of oral feeding skills [20] or the delayed desensitization of the gag reflex [24]. Children with oral feeding disorders, including extreme levels of pickiness, are more likely to develop neurodevelopment disorders, possibly due to a common underlying genetic susceptibility [25]. At a population level, however, delayed introduction to textured foods may not always be informed by the individual child’s predispositions according to a responsive feeding framework [26–28]. In France, mothers seem to be particularly reluctant to introduce more complex textures due to fear of choking, although their children respond favourably to such textures [19, 29, 30].

However, a general delayed introduction to textured foods beyond the critical window of exposure, and beyond the individual child’s predisposition may relate to several neurodevelopmental domains, which culminate in overall effects on neurodevelopment. Several mechanisms may be involved in the aforementioned processes. Recent evidence from a rat study has shown that a soft diet during weaning can induce changes in jaw movements that are represented in cortical motor areas [31], corroborating earlier evidence in studies in other animals [32]. In addition, masticatory hypofunction resulting from a soft diet was found to induce neuronal changes in the thalamus of the growing rat, potentially affecting brain function [33]. Thus, aspects of neurodevelopment, as they manifest in oral/gross motor skills as well as brain development, seem to be intertwined with feeding increasingly complex textures [17]. Such links may extend to language capacities and cognition, given the development of oral muscles and motor skills involved in both eating and language [31, 34–37]. However, no research to date has investigated the relation between the introduction of textured foods in early infancy and aspects of neurodevelopment in early childhood.

The present study seeks to understand the associations between exposure to food texture through the introduction to food pieces in infancy and neurodevelopment up to age 3.5 years in the French ELFE birth cohort. Our hypothesis is that timely introduction to food texture favorably relates to overall neurodevelopmental outcomes at the various follow-up points, which pertain to language, gross and fine motor skills and cognition. In addition, different foods describe increasingly complex textures, as fruit and vegetables can be easily manipulated into more simple textures including lumps, while meat pieces may be characterized by more complex textures.

## Methods

### Study design

This study is based on data from the nationwide Étude Longitudinale Française depuis l’Enfance (ELFE) cohort in France. Families (*n* = 18329) were recruited at birth from 320 maternity units in metropolitan France over four recruitment waves comprising 25 selected days in 2011. The sampling strategy was stratified based on the size of the units [38].

### Participants

**Fig. 1:**
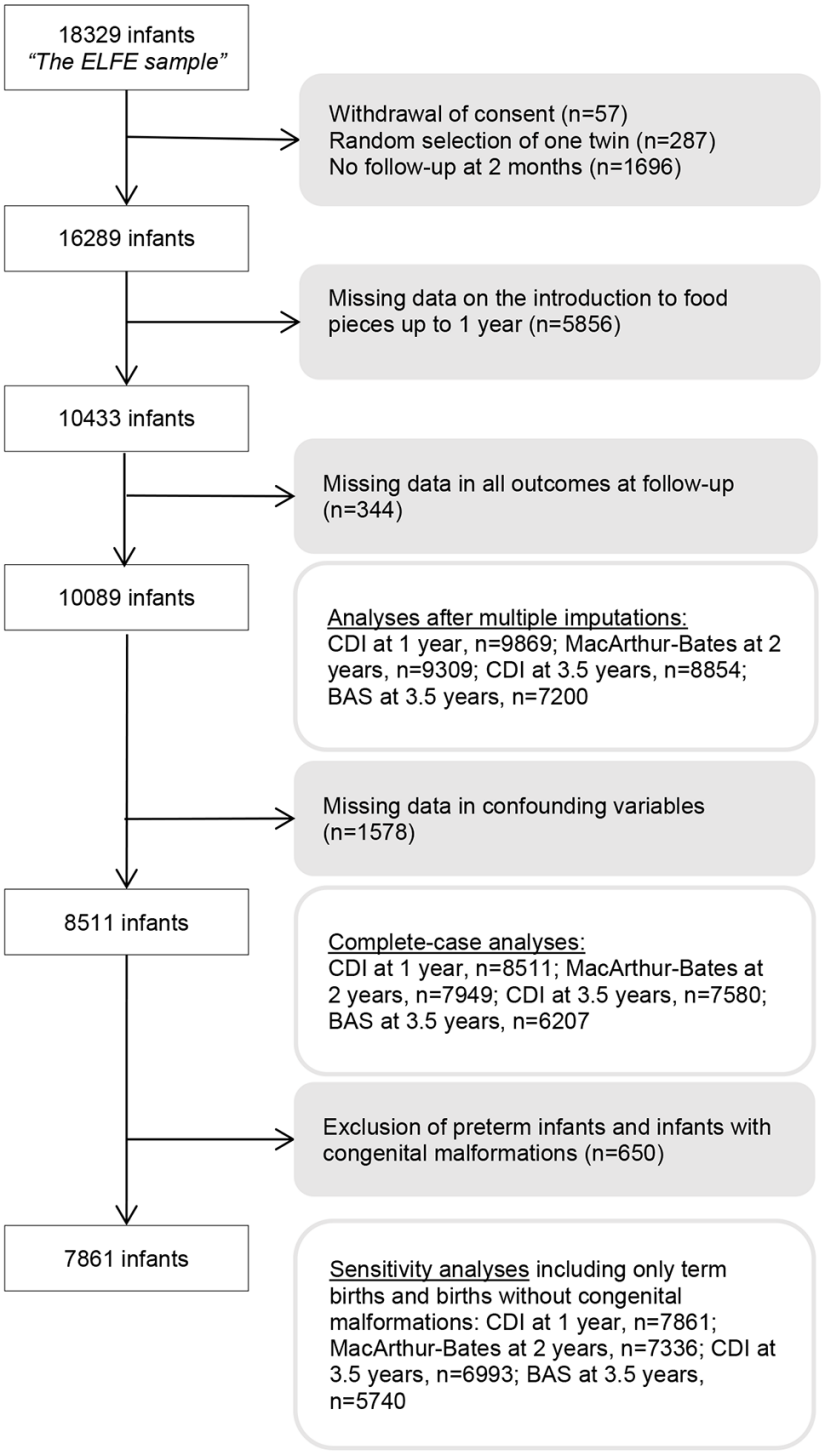
Flowchart outlining the process for selecting the analytical sample

Eligibility was based on single or twin live births at ≥ 33weeks of gestation, a mother ≥ 18 years old, and no plans to leave metropolitan France within 3 years. Follow-up assessments were performed at 1, 2 and 3.5 years using parent-reported data via phone and assessments by a research assistant. The selection of the analytical sample (*n* = 8511) is shown in Fig. 1. Table S1 (Supplementary material) provides a comparison between the excluded and complete-case samples.

### Measurement

#### Food piece introduction and complementary feeding practices

Parents prospectively reported on their complementary feeding practices on a monthly basis between 3 and 10 months [38–40]. Introduction to food pieces was assessed monthly between 2 and 10 months follow-up and parents indicated the frequency of offering mashed fruits/vegetables (assessed from 3 months onward) or pieces of meat (assessed from 6 months onward) between two consecutive monthly assessment follow-up time points. Based on these detailed parental reports, introduction to food pieces was estimated as the occasional (at least once) provision of pieces of mashed fruits/vegetables or pieces of meat, whichever came first. Introduction to food texture was classified according to three categories (along with their respective distribution in the sample) [1]: (1) “early”, before 8 months (39.3%); (2) “intermediate”, between 8 and 10 months (34.3%); and (3) “late”, after 10 months (26.4%). The classification is based on the evidence that introduction of at least minimally textured foods, i.e. mashed fruits or vegetables, can be beneficial for food acceptance and establishing eating habits [1].

In addition, we further explored distinct exposures to food texture according to the introduction to at least mashed fruits/vegetables with lumps (or small pieces of fruits/vegetables) and small meat pieces. The aforementioned general exposure category for introduction to food texture, according to both introduction of at least mashed fruits/vegetables or meat pieces, to a large degree corresponds to the introduction of fruits/vegetables specifically. However, the classification of food texture introduction according to fruits/vegetables cannot really differentiate between increasingly complex textures from mashed lumpy foods and those offered in small pieces. Therefore, we included separate analyses for introduction to small meat pieces specifically. Given the overall late introduction to such textures in the first year the variables was categorised as follows: (1) “early”, before 10 months; (2) “late”, after 10 months.

Moreover, age at introduction to complementary feeding and any breastfeeding duration were calculated, as explained in previous studies [39, 41]. Breastfeeding duration was classified as follows: never, up to 1 month, between 1 and 3 months, between 3 and 6 months, between 6 and 9 months, beyond 9 months. Age at introduction to complementary feeding was classified into three categories (earlier than 4 months, between 4 and 6 months, after 6 months).

#### Neurodevelopmental assessment

Child cognitive and motor skills were assessed through various instruments. While the Child Development Inventory (CDI, at 1 and 3.5 years) [42, 43] and the MacArthur-Bates Communicative Development Inventory (MB-2, at 2 years) [44] were parent-reported via phone consultations, the Pictures Similarities subscale of the British Ability Scale was administered by research assistants during home visits (PS-3.5, at 3.5 years) [45]. Briefly, the CDI assesses the number of developmental achievements of children aged 15–72 months in eight domains (social, self-help, gross motor, fine motor, expressive language, language comprehension and letter and numeracy knowledge). Response options for each item were yes (1) or no (0) according to the child having achieved the described ability or not. The composite score ranges from 0 to 50 at 1 year and from 17 to 62 at 3.5 years (for the latter, the assumption is that older infants have demonstrated achievements corresponding to the earlier assessment at 1 year, which defines the lowest limit). The MB-2 score is a measure of expressive language acquisition ranging from 0 to 100, and it corresponds to the number of words a child is able to pronounce spontaneously from a list of 100 words. PS-3.5 assesses the child’s nonverbal reasoning and visual perception and analysis; the score ranges from 10 to 119 [46]. For all instruments, higher scores indicate higher individual capacity in the neurodevelopmental domain.

The developmental quotient at 3.5 years (DQ-3.5) was used in order to examine the present hypotheses on child neurodevelopment from a clinical perspective considering the developmental achievements of the child at 3.5 years, i.e. developmental age of the child, in relation to their chronological age. The DQ-3.5 was estimated as the ratio of the child’s developmental age according to the continuous CDI-3.5 score, as defined in the French norms for the CDI [43], to the child’s chronological age (DQ-3.5 = developmental age/chronological age x 100). A DQ-3.5 < 90 indicates a high risk of developmental delay [43]. Thus, DQ-3.5 was used as a binary variable for further assessments, which pertain to clinical interpretations.

#### Perinatal, family and demographic variables

Family and perinatal characteristics were assessed by trained interviewers at the maternity ward, and they were complemented by data on the newborn from medical records. Phone interviews at 2 months and 1 year postpartum yielded additional information regarding the families recruited in the study [38].

The family and demographic characteristics of interest were as follows: maternal age at delivery (< 25 years, 25–29 years, 30–34 years, ≥ 35 years), maternal employment during pregnancy (employed, unemployed, out of the labour force, i.e., housewife, retired, students), maternal education level (up to upper secondary, intermediate, 3-year university degree, at least 5-year university degree), maternal migration history (immigrant, descendant of at least one immigrant, rest of population), household income per consumption unit (≤ 1110 €/month, 1111–1500 €/month, 1501–1944 €/month, 1945–2500 €/month, > 2500 €/month), mother smoking during pregnancy (never smoker, smoker only before pregnancy, smoker only in early pregnancy, smoked throughout pregnancy), number of older siblings in the household (first child, second child, at least third child), collective care attendance (first attendance by 4 months, first attendance between 4 and 6 months, first attendance between 6 and 12 months, no collective care up to 12 months).

The region of residence (Paris region, North, East, Paris Basin – East, Paris Basin – West, West, Southwest, Southeast, Mediterranean) and the residence area size (urban, rural area) were determined from the postal code of the family residence. At the 1-year interview, the mother indicated the frequency (rarely/never/sometimes, often) of some activities with their child: playing, reading books, drawing, speaking, tickling/massage. The modal value of these activities was used to estimate a maternal stimulation score.

Data were collected on the following perinatal and infant characteristics: child sex (boy, girl), birth weight category (small for gestational age, adequate for gestational age, large for gestational age), gestational age (in weeks), maternal pre-pregnancy BMI status (< 18.5 kg/m², 18.5–24.9 kg/m², 25.0–29.9 kg/m², ≥ 30.0 kg/m²).

Confounding variables were identified in the literature and were selected through the directed acyclic graph method [47], as detailed in the Supplementary Material (Figure S1). While a full description of data collected within the ELFE study is described in the study protocol [38], Table S2 in the Supplementary Material provides a full list of the confounding variables and, among those, the modalities of the categorical variables among those.

### Statistical analyses

According to available data on neurodevelopmental outcomes, the main analyses were performed on a complete-case basis (Fig. 1).

Differences across the three categories of introduction to food texture were assessed using one-way ANOVA for continuous variables and chi-squared tests for categorical variables. Similarly, differences between excluded and included families and children were assessed using Student’s t test and chi-squared test for continuous and categorical variables, respectively.

To allow comparison across models, the continuous neurodevelopmental scores, i.e. CDI-1, MB-2, CDI-3.5 and PS-3.5, were standardized before analyses.

Linear regression models were used to examine the unadjusted and adjusted associations between the age at introduction to food pieces and continuous neurodevelopment scores. The variables that were considered in the adjusted models were the following: maternal demographic and health characteristics (maternal age at delivery, maternal employment during pregnancy, maternal education level, maternal migration history, household income per consumption unit, mother smoking during pregnancy, maternal pre-pregnancy BMI status, number of older siblings in the household, residence area size), child characteristics (child sex, birth weight category, gestational age, collective care attendance, parental stimulation) and aspects of early feeding (any breastfeeding duration and age at introduction to complementary feeding). Three additional variables were considered according to the study design and recruitment processes, i.e. recruitment wave (four waves), maternity unit size (five strata based on yearly number of deliveries) and region of residence according to postal codes (nine regions in metropolitan France spreading North-South and West-East). According to the respective diagnostics, collinearity was not an issue in such analyses, i.e. the variance inflation factor did not exceed 2.6.

Moreover, we run a second adjusted model for neurodevelopmental scores at ages 2 and 3.5 years, which was adjusted for the CDI-1 summary score, to address reverse causation. We also considered separate adjusted models for the introduction of fruit/vegetable pieces and meat pieces as exposure variables. Similarly, logistic regression models were used to analyze the discrete developmental outcome (DQ-3.5 < 90).

Sensitivity analyses were performed including subsamples and procedures considering biases. First, analyses included both term births and births without any type of congenital malformations (*n* = 7861) based on availability of neurodevelopmental data at the respective time points. Preterm birth was defined according to gestational age (i.e., born before 37 weeks) [38, 48]. Codes for congenital malformations (e.g. nervous and circulatory systems, chromosomal abnormalities) were based on the International Statistical Classification of Diseases and Related Health Problems, 10th Revision [49]. Second, we performed weighted analyses to deal with selection and attrition bias. Weighting was applied on complete-case subsamples for each neurodevelopmental score, which included calibration using margins from the state register’s statistical data and the 2010 French National Perinatal study [50] on the following variables: age, region, marital status, migration status, level of education, and primiparity according to the ELFE report on weighting national survey data [51]. A specific weighting was calculated for each subsample included in the complete-case analyses at 1, 2, and 3.5-year follow-up points. Third, multivariate imputation by chained equations was performed on the assumption that data were missing at random using the fully conditional specification method (MI procedure) to address missing data on confounding factors. We imputed categorical variables with multinomial logistic regression models, ordinal or binary variables with logistic regression models, and continuous variables with linear regression models. Ten independent and complete datasets were generated and pooled outcome estimates and their 95% confidence intervals were calculated (MIANALYZE procedure) [52].

We performed additional analyses considering the specific subdomains of the composite scores of the CDI at 1 and 3.5 years follow-up. The six domain-specific sub-scores, however, did not follow a normal distribution; thus, they were divided into quartiles. A low developmental sub-score was defined according to the lowest quartile, which was compared with the reference group, as characterised by the three upper quartiles. Therefore, logistic regressions models were fitted and they were adjusted as described earlier in this section (Table S5 in Supplementary material).

All analyses were carried out using SAS v9.4 (SAS Institute Inc., Cary, NC, USA). Significance was set at *p* < 0.05.

## Results

Compared with children who were included in the main analyses, those excluded were less frequently born to mothers with high education level (15.9% vs. 23.8% have at least 5 years of university education), with no migrant background (72.6% vs. 85.3%) and with employment (62.1% vs. 79.8%). Data are shown in Supplementary material (Table S1).

Table 1 describes family, sociodemographic/health parameters, and infant feeding practices across the three groups of time to introduction to food pieces (i.e., early/before 8 months, 39.3%; intermediate/between 8 and 10 months, 34.3%; late/after 10 months, 26.4%). In particular, compared with children introduced to food texture at intermediate or late follow-up points, respectively, those introduced early were more frequently born to mothers with a lower education level (33.1% vs. 26.2% and 26.2%, respectively), with migrant background (8.9% vs. 5% and 3.4% respectively) and to households with low income (17.5% vs. 12.7% and 11.3%, respectively).

**Table 1:** Characteristics and differences across groups of increasing age at introduction to food pieces, complete-case (*n* = 8511)

	Age at introduction to food pieces		
	Before 8 months; “early”	Between 8 and 10 months “intermediate”	After 10 months “late”
N	**3341**	**2922**	**2248**
**Socio-demographic and health variables**			
**Maternal age at delivery**, % (n) **			
< 25 years	7.7% (256)	4.8% (139)	3.6% (81)
25–29 years	30.2% (1008)	29.2% (853)	28.7% (645)
30–34 years	39.3% (1314)	42.1% (1229)	44.5% (1000)
≥ 35 years	22.8% (763)	24.0% (701)	23.2% (522)
**Maternal employment during pregnancy**, % (n) **			
Employed	75.3% (2515)	81.9% (2393)	83.7% (1881)
Unemployed	11.5% (383)	9.4% (276)	8.4% (188)
Out of the labour force (i.e., housewife, retired, students)	13.3% (443)	8.7% (253)	8.0% (179)
**Maternal education level**, % (n) **			
Up to upper secondary	33.1% (1107)	26.2% (765)	26.2% (590)
Intermediate	23.9% (800)	27.2% (795)	25.2% (566)
3-y university degree	20.2% (674)	22.5% (657)	23.5% (528)
At least 5-y university degree	22.7% (760)	24.1% (705)	25.1% (564)
**Maternal migration history**, % (n) **			
Born to French parents	80.2% (2680)	87.3% (2550)	90.2% (2028)
Descendant of at least one immigrant	10.9% (363)	7.8% (227)	6.4% (143)
Immigrant	8.9% (298)	5.0% (145)	3.4% (77)
**Household income per consumption unit**, % (n) **			
≤ 1110 €/month	17.5% (584)	12.7% (370)	11.3% (254)
1111–1500 €/month	28.8% (963)	26.9% (785)	27.5% (619)
1501–1944 €/month	26.5% (886)	28.9% (844)	29.3% (658)
1945–2500 €/month	17.5% (584)	19.7% (575)	21.4% (482)
> 2500 €/month	9.7% (324)	11.9% (348)	10.5% (235)
**Residence area**, % (n) **			
Urban	77.6% (2594)	74.5% (2178)	74.2% (1667)
Rural	22.4% (747)	25.5% (744)	25.8% (581)
**Mother smoking during pregnancy**, % (n) *			
Never smoker	58.0% (1937)	58.9% (1715)	59.8% (1341)
Smoker only before pregnancy	25.9% (865)	26.8% (780)	26.7% (598)
Smoker only in early pregnancy	3.6% (121)	3.5% (103)	3.3% (73)
Smoker throughout pregnancy	12.5% (418)	10.8% (313)	10.2% (229)
**Mother BMI status**, % (n) *			
< 18.5 kg/m²	6.7% (224)	6.9% (201)	6.7% (150)
18.5–24.9 kg/m²	66.7% (2229)	67.8% (1982)	67.7% (1522)
25.0–29.9 kg/m²	17.3% (578)	15.9% (465)	18.1% (406)
≥ 30.0 kg/m²	9.3% (310)	9.4% (274)	7.6% (170)
**Parental stimulation**^**a**^, % (n) **			
Often	70.3% (2348)	67.7% (1978)	64.3% (1445)
Sometimes	27.8% (929)	30.6% (893)	34.4% (774)
Rarely or never	1.9% (64)	1.7% (51)	1.3% (29)
**Child sex**, % (n) *			
Boy	51.8% (1729)	51.1% (1494)	50.0% (1123)
Girl	48.2% (1612)	48.9% (1428)	50.0% (1125)
**Birth weight category**^**b**^, % (n) *			
Small for GA	7.9% (263)	9.7% (284)	9.3% (208)
Adequate for GA	81.7% (2731)	79.6% (2325)	80.3% (1805)
Large for GA	10.4% (347)	10.7% (313)	10.5% (235)
**Gestational age in weeks**, Mean (SD) **	39.4(1.4)	39.3(1.4)	39.3(1.4)
**Number of older siblings in the household**, % (n) **			
First child	48.4% (1618)	45.8% (1338)	42.0% (945)
Second child	34.8% (1161)	38.5% (1124)	42.1% (947)
At least third child	16.8% (562)	15.7% (460)	15.9% (356)
**Collective care attendance**, % (n) **			
No collective care up to 12 months	54.6% (1824)	47.8% (1398)	47.3% (1064)
First attendance by 4 months	22.0% (736)	27.0% (789)	27.8% (624)
First attendance between 2 and 6 months	10.3% (345)	12.8% (375)	13.2% (297)
First attendance between 6 and 12 months	13.0% (436)	12.3% (360)	11.7% (263)
**Variables on early child feeding practices**			
**Any breastfeeding duration in months**, Mean (SD) **			
Never	21.3% (712)	22.7% (664)	27.4% (615)
Up to 1 month	16.2% (541)	15.8% (462)	15.8% (355)
Between 1 and 3 months	16.0% (536)	17.1% (500)	15.4% (347)
Between 3 and 6 months	18.5% (618)	20.3% (594)	19.0% (426)
Between 6 and 9 months	11.5% (385)	12.7% (372)	12.5% (281)
Beyond 9 months	16.4% (549)	11.3% (330)	10.0% (224)
**Age at introduction to complementary feeding**, % (n) **			
Earlier than 4 months	26.6% (888)	16.7% (487)	13.4% (302)
Between 4 and 6 months	63.3% (2116)	67.5% (1973)	64.9% (1460)
After 6 months	10.1% (337)	15.8% (462)	21.6% (486)
**Neurodevelopmental assessments**			
**CDI-1**,** Mean (SD)**	37.6(5.3)	36.5(5.4)	35.1(5.7)
**MB-2**,** Mean (SD)**	73.8(24.5)	72.9(25)	70.7(25.5)
**CDI-3.5**,** Mean (SD)**	54.1(5)	53.6(4.9)	53(5.4)
**PS-3.5**,** Mean (SD)**	68(15.4)	67.5(15.9)	67.4(15.6)
**DQ < 90**,** % (n)**	11.2% (325)	11.9% (313)	16.5% (336)

^a^ Parental stimulation was defined according to the frequency of activities (e.g. drawing, playing) with the child, as reported by mothers at the 1-year follow-up

^b^ Size at gestational age is classified according to birth weight

*p-value for comparisons across exposure categories >0.05; one-way ANOVA for continuous variables and chi-squared tests for categorical variables

** p-value for comparisons across exposure categories <0.05; one-way ANOVA for continuous variables and chi-squared tests for categorical variables

Moreover, Table 1 describes summary statistics of neurodevelopmental outcomes across the three categories of exposure. As for the statistics of the total analytical sample (data not shown), the mean neurodevelopmental scores are (mean; SD): CDI-1 (36.6; 5.6), MB (72.6; 25), CDI-3.5 (53.6; 5.1), PS-3.5 (67.7; 15.6), while 12.8% of children are considered at risk for developmental delay.

All analyses were based on standardized summary neurodevelopmental scores, i.e. CDI-1, MB-2, CDI-3.5 and PS-3.5. The reported estimates (along with 95% CIs) correspond to differences in SD regarding the continuous neurodevelopmental outcomes.

The associations between age at introduction to pieces and neurodevelopment showed a consistent pattern (Table 2). In complete-case analyses, compared to early introduction to food pieces, intermediate and late introductions were associated with lower scores on CDI-1, MB-2, and CDI-3.5. In contrast, we failed to detect any associations with PS-3.5. While children who had been introduced to pieces late, i.e. beyond 10 months, compared to those introduced to pieces early, were more likely to be at risk for developmental delay at 3.5 years (OR 1.62, 95% CI 1.36;1.94), no such association was detected for intermediate introduction.

**Table 2:** Adjusted estimates (95% CI) and ORs (95% CI) of neurodevelopmental outcomes in terms of age at introduction to food pieces. Total sample and sub-samples including both term births and births without congenital malformations, complete-case analyses

		Neurodevelopmental scores (standardised continuous)				At risk for developmental delay (binary)
		CDI-1	MB-2	CDI-3.5	PS-3.5	DQ-3.5 < 90
		Estimate [95% CI]	Estimate [95% CI]	Estimate [95% CI]	Estimate [95% CI]	OR [95% CI]
*Total sample*,* adjusted*^*a*^		*N* = 8511	*N* = 7949	*Ν = 7580*	*Ν = 6207*	*N* = 7580
	Before 8 months	0 [Ref.]	0 [Ref.]	0 [Ref.]	0 [Ref.]	1 [Ref.]
	Between 8 and 10 months	**-0.12 [-0.17; -0.08]**	**-0.06 [-0.11; -0.01]**	**-0.08 [-0.12; -0.03]**	-0.04 [-0.10; 0.02]	1.12 [0.94; 1.33]
	After 10 months	**-0.35 [-0.40; -0.30]**	**-0.15 [-0.20; -0.10]**	**-0.18 [-0.23; -0.13]**	-0.04 [-0.10; 0.02]	**1.62 [1.36; 1.94]**
*Total sample*,* adjusted*^*b*^		*Ν/Α*	*N* = 7949	*Ν = 7580*	*Ν = 6207*	*Ν = 7580*
	Before 8 months	Ν/Α	0 [Ref.]	0 [Ref.]	0 [Ref.]	1 [Ref.]
	Between 8 and 10 months	Ν/Α	-0.02 [-0.07; 0.03]	-0.03 [-0.08; 0.01]	-0.02 [-0.08; 0.03]	1.03 [0.86; 1.23]
	After 10 months	Ν/Α	-0.03 [-0.08; 0.02]	**-0.05 [-0.10; 0.00]**	0.01 [-0.06; 0.07]	**1.24 [1.03; 1.50]**
*Births full-term and no malformations* ^*a*^		*N* = 7861	*N* = 7336	*N* = 6993	*N* = 5740	*N* = 6993
	Before 8 months	0 [Ref.]	0 [Ref.]	0 [Ref.]	0 [Ref.]	1 [Ref.]
	Between 8 and 10 months	**-0.11 [-0.16; -0.06]**	**-0.05 [-0.10; 0.00]**	**-0.05 [-0.11; 0.00]**	-0.03 [-0.09; 0.02]	1.08 [0.90; 1.30]
	After 10 months	**-0.35 [-0.40; -0.30]**	**-0.14 [-0.20; -0.09]**	**-0.17 [-0.23; -0.12]**	-0.03 [-0.09; 0.03]	**1.61 [1.34; 1.94]**
*Total sample*,* unadjusted*		*N* = 8511	*N* = 7949	*Ν = 7580*	*Ν = 6207*	*N* = 7580
	Before 8 months	0 [Ref.]	0 [Ref.]	0 [Ref.]	0 [Ref.]	1 [Ref.]
	Between 8 and 10 months	**-0.20 [-0.25; -0.15]**	-0.04 [-0.09; 0.02]	**-0.08 [-0.14; -0.03]**	-0.03 [-0.09; 0.02]	1.07 [0.91; 1.27]
	After 10 months	**-0.45 [-0.50; -0.39]**	**-0.12 [-0.18; -0.07]**	**-0.20 [-0.26; -0.15]**	-0.04 [-0.1; 0.02]	**1.57 [1.33; 1.85]**

CDI-1: Child Development Inventory at 1 year; MB-2: McArthur-Bates Communicative Development Inventory at 2 years; CDI-3.5: Child Development Inventory at 3.5 years; PS-3.5: Pictures Similarities subscale of the British Ability Scale at 3.5 years; DQ-3.5<90: Developmental Quotient (developmental age/chronological age x 100) below 90 indicating a possible risk for developmental delay

Estimates (95% CI) for CDI-1, MB-2, CDI-3.5 and PS-3.5 were calculated using linear regression models

ORs (95% CI) for DQ-3.5<90 were calculated using binary logistic regression models

^a^ Adjustments considered the following confounding variables and covariates: maternal age at delivery, maternal employment, maternal smoking during pregnancy, maternal education level, maternal migration history, household income per consumption unit, residence area size, maternal BMI status before pregnancy, parental stimulation, child sex, child birth weight category, child gestational age, number of older siblings in the household, collective care attendance, breastfeeding duration and age of introduction to complementary feeding, along with recruitment wave, maternity unit and region of residence. Age at neurodevelopmental assessment was further considered, when applicable, i.e. in the linear regression models for CDI-1, MB-2, CDI-3.5 and PS-3.5

^b^ Further adjustment for CDI-1 in models for later assessments; N/A for the CDI-1 outcome

However, adjusting for CDI-1 reduced the strength of certain estimates (by 70–80% for MB-2 and CDI-3.5 and by 20% for the discrete DQ < 90), but the associations remained significant for CDI-3.5 and DQ-3.5 < 90 (Table 2).

Results were similar after exclusion of preterm infants and those with congenital malformations (Table 2), in imputed and weighted analyses (Table S3 in Supplementary material). In complete-case anlayses, similar associations were found when considering introduction to food pieces in terms of fruits/vegetables and meat separately (Table S4 in Supplementary material).

## Discussion

For the first time, using longitudinal data, we report on negative associations between later introduction to textured food/pieces and neurodevelopmental outcomes at ages 1, 2 and 3.5 years (continuous scores and risk for developmental delay) in the general population. In particular, delayed introduction to food texture, beyond 10 months, was consistently associated with lower composite neurodevelopmental scores at 1 and 3.5 years (CDI-1 and CDI-3.5), with a lower score for language acquisition at 2 years (MB-2), and with a higher risk for developmental delay at 3.5 years (DQ-3.5 < 90), with the latter highlighting possible clinical aspects of the findings. Despite the attenuated associations after adjusting for the composite score at 1 year, the associations held regarding the neurodevelopmental outcomes at 3.5 years, i.e. the composite score and the risk for developmental delay. The findings were confirmed in sensitivity analyses, which included a low risk subsample, weighted data in order to address attrition and selection bias as well as imputed data in order to address missing data.

While there have been studies to support the relevance of timely introduction to food pieces for food preferences [9, 10, 18, 53], little research has addressed our specific question. To our knowledge, one recent study provides evidence supporting our findings. Webber et al. examined language development (language production and comprehension) using the same instrument, i.e., the MacArthur Bates Communicative Development Inventory, in relation to complementary feeding [34]. It was shown that a baby-led approach emphasizing the consumption of family finger foods was associated with better language outcomes. It is noteworthy that family foods are more likely to preserve complex textures, as they are not adapted for infants, and that age at introduction to solids was not associated with language outcomes per se [34]. In the same study, the authors implicated the development of motor skills (independent self-feeding), which was shown to facilitate feeding finger foods [34]. Therefore, they linked gross and oral motor skills to language development through the ability to handle complex textures inasmuch as similar muscles and brain areas are involved in the control of these behaviors [35, 37]. However, the cross-sectional nature of the study did not allow for a follow-up on neurodevelopment over time or for disentangling the temporal associations across possible exposure to more complex textures (through family foods), motor skills and language skills.

Our most consistent findings in terms of the direction and size of the effects refer to the binary outcome defining risk for developmental delay. While these analyses suggest a consistent unfavorable association with late introduction to food pieces, they do not present any association in terms of intermediate introduction. This is in contrast to the unfavorable associations with increasing age at introduction to food pieces shown for continuous outcomes. This discrepancy suggests that late introduction to pieces is adversely associated with neurodevelopment, yet intermediate introduction may simply describe a typical variation in the population and is not linked to clinical outcomes requiring further consideration. Moreover, the findings are in line with the ESPGHAN, which recommends the introduction *“of lumpy foods by 8 to 10 months at the latest”* [1].

In addition, it is important to highlight that while the use of the binary outcome describing the risk of developmental delay is of clinical relevance, it is not a clinical outcome per se. Thus, in order to provide some framework for the interpretation of the magnitude of effects, in light of the outcome distribution, we hereby compare the size of the effect of the introduction to texture to that of other variables with well-established links to aspects of neurodevelopment. Regarding language expression, the average child expressed about 10 words more at 2 years of age than the average child in another French study that was smaller in scope, though the latter study showed higher dispersion [54]. We found that the average child introduced to texture earlier during the first year expressed about three words more at 2 years of age, as compared to having being introduced to texture later. This is comparable to the associations found in the French EDEN mother-child cohort regarding the same language score at 2 years of age for ever breastfeeding compared to children who had never been breastfed [55] as well as for over 2-hour per day screen time compared to no screen time [54]. Last, the effect size of texture introduction on the global score for child neurodevelopment is higher than that for maternal diet quality based on data from the same cohort [46].

While our findings do not substantiate a cause-effect relation and reverse causation cannot be fully excluded, we may argue that a temporal association between timely introduction to food pieces and neurodevelopmental outcomes is, to some extent, possible. This relates to the study design and sensitivity analyses. First, by 8 months old or even earlier [11, 17], typically developing infants are able to handle food pieces/textures as assessed in this study, i.e., feeding (at least) crushed/mashed vegetables or fruits, which represent relatively simple textures defining the transition between pureed and solid family foods [12, 30]. Indeed, the present analyses are based on data from a birth cohort covering the general population in France (i.e., the majority of infants following typical developmental trajectories). Moreover, the main findings were confirmed in models including a subsample with no background conditions favoring slower development (such as being born preterm or bearing a congenital malformation) and in models adjusted for the earliest available developmental assessment at 1 year. However, in the latter analyses, effect sizes were attenuated. To better disentangle the temporal associations between early feeding practices, including sensory aspects, and neurodevelopment, repeated assessments during infancy are crucial, while accounting for the early sensory profiles of the children [56, 57].

Concurrently, we need to consider parental awareness and concerns about child eating and developmental cues. A critical window (certainly before 10 months) exists for the exposure to food textures, which could further facilitate oral motor skills and other neurodevelopmental outcomes [8, 17, 20, 31, 34]. Maternal responsiveness to child feeding cues is an active research area building on parenting research [26–28, 58]. Responsive feeding can expand our understanding of introduction to texture beyond the comprehensive assessment of early feeding practices (as performed in the present study) through the inclusion of parental (at least maternal) experiences and motives. For example, autism spectrum disorder is characterised by sensory reactivity, which may prompt parents to delay the introduction to food texture [59, 60]. Thus, it would be relevant to further explore the influence that early sensory indicators of such neurodevelopmental disorders may have on early feeding practices, and in particular on the timing of introduction to food pieces. However, no data were available within the ELFE cohort on the reasons or motives that prompted the introduction (or delayed introduction) of food pieces/texture throughout the first year of life. Nevertheless, French studies including elaborate assessments of food texture acceptance suggest that early feeding experiences are not solely based on child developmental readiness or eating cues but also on maternal readiness [11, 18]. First, mother-reported infant acceptance of textured food (even rough purees as a transitional textured food) was found to be, to some extent, a function of mothers’ feelings toward offering such food and actually offering it [18]. In addition, survey data highlighted that one third of French parents were reluctant to introduce food pieces [18]. Interestingly, the proportion of reluctant parents was higher for infants and children older than 8 months old [18]. Although the types of foods were not specified in the study, such observations may simply showcase that food rejection, especially at first, is a normal part of complementary feeding, and thus a common experience among parents when it is first attempted [12]. A pilot intervention confirmed the difficulty of altering parental food piece introduction practices, especially for textures that are perceived as more challenging, such as large and/or hard pieces [19]. It is noteworthy that children are often able to handle textures that are more complex than those actually offered to them [11], and familiarity through exposure can facilitate the process [12]. Taken together, these findings suggest that introduction to food pieces may pose specific challenges that are not always informed by the infant’s developmental readiness, and further research is needed to understand such motives and offer appropriate guidance [9, 10].

### Strengths and limitations

The present study is based on a large dataset from the representative and nationwide ELFE cohort in France [38]. The large sample size and assessments of background socio-demographic and other characteristics that describe child development and early feeding practices both ensure power and facilitate controlling for relevant variables. Nevertheless, residual confounding may not be overruled. Moreover, the study was based on comprehensive assessments of feeding practices over the first year [39, 61] as well as valid assessment instruments for neurodevelopmental outcomes [42–45]. However, our findings need to be interpreted in the context of existing limitations. First, the sample that provided the estimates for our main analyses was more privileged than the total sample in terms of an array of socio-demographic characteristics, such as income, education and migrant status. Nevertheless, the design of the ELFE cohort is characterised by random sampling of the maternity units that facilitated recruitment and by the availability of data regarding non-respondents in the cohort. This approach allows for the evaluation of weighted data, which can provide insights into the generalisability of the main findings to the target population, i.e. births in France in 2011. Sensitivity analyses were performed in order to address attrition/selection bias, which confirmed the main findings. Similarly, sensitivity analyses based on imputed data in order to address bias due to missing data, confirmed the main findings. Moreover, we showed inconsistent associations between parent-reported assessments and the PS-3.5 administered during home visits. Bias may have been introduced due to the mode of data collection (home visits); alternatively, PS-3.5 assesses cognitive capacities, which may have less pronounced links to developmental skills related to food texture, such as motor and language skills [34, 35]. The main limitation, as already discussed, is that analyses do not allow a causal mechanism to be proposed.

## Conclusions

Our findings demonstrate that the timing of introduction to food pieces during complementary feeding is associated with parent-reported neurodevelopmental outcomes in early childhood up to 3.5 years. In particular, late introduction of food pieces (> 10 months) was associated with lower neurodevelopmental scores. Although there is a biological basis to support such an association, future research should include a more comprehensive assessment of early neurodevelopmental readiness and ascertain the findings while limiting issues with reverse causation.

## Electronic supplementary material

Below is the link to the electronic supplementary material.

Supplementary Material 1

## Data Availability

The datasets generated and analysed during the current study are not publicly available for reasons of privacy for the participants. Established researchers who would like access to the data from the Elfe cohort study can request them to the Committee of Access to the Data from the Elfe cohort on the website of the survey: www.elfe-france.fr.

## Abbreviations

ESPGHAN: the European Society for Paediatric Gastroenterology, Hepatology, and Nutrition
ELFE: Étude Longitudinale Française depuis l’Enfance
DQ: Developmental Quotient
CDI: Child Development Inventory
MB: MacArthur-Bates Communicative Development Inventory
PS: Pictures Similarities subscale of the British Ability Scale
