# Supplementary material for: Timing of food pieces introduction and neurodevelopment: findings from a nationwide birth cohort
Source: Int J Behav Nutr Phys Act. 2024 Oct 16;21:118. doi: 10.1186/s12966-024-01669-5 (PMC11481772; doi:10.1186/s12966-024-01669-5)
Supplement: Supplementary file 1 — Supplementary Material 1 [file 12966_2024_1669_MOESM1_ESM.docx]

# Supplementary material for manuscript Somaraki et al. “Timing of food pieces introduction and neurodevelopment: findings from a nationwide birth cohort”

Table S1. Background characteristics of the analytical sample and the excluded sample.

Table S2. Confounding variables: categorical variables (with their modalities) and continuous variables.

Figure S1. Direct Acyclic Graph (DAGs) applied to adjusted analyses throughout the manuscript using Daggity v 3.0^2^.

Table S3. Adjusted estimates (95% CI) and ORs (95% CI) of neurodevelopmental outcomes in total sample, weighted and imputed analyses.

Table S4. Adjusted estimates (95% CI) and ORs (95% CI) of neurodevelopmental outcomes in separate models for introduction to food pieces in terms of fruits/vegetables and meat, respectively, complete-case analyses.

Table S5. Adjusted ORs of having a poor developmental sub-score across categories of increasing age at introduction to food pieces, complete-case analyses at 1-year follow-up (n=8511) and at 3.5 years follow-up (n=7580).

## Part A: Differences between the analytical sample and excluded sample

Table S1 compares the analytical sample to the sample excluded from analyses according to available data on socio-demographic and health characteristics and infant feeding practices.

Table S1. Background characteristics of the analytical sample and the excluded sample.

|  | | **Excluded from the analytical sample (n=9474)** | **Complete-case sample (n=8511)** | **P-value** |
| --- | --- | --- | --- | --- |
| Socio-demographic and health | | | | |
| **Maternal age, % (n)** | |  |  | <0.001 |
|  | <25 years | 15.4% (1451) | 5.6% (476) |  |
|  | 25-29 years | 30.9% (2911) | 29.4% (2506) |  |
|  | 30-34 years | 30.5% (2873) | 41.6% (3543) |  |
|  | ≥35 years | 23.2% (2185) | 23.3% (1986) |  |
| **Maternal employment, % (n)** | |  |  | <0.001 |
|  | Employed | 62.1% (4737) | 79.8% (6789) |  |
|  | Unemployed | 14.7% (1123) | 10.0% (847) |  |
|  | Out of the labour force (i.e. housewife, retired, students) | 23.2% (1770) | 10.3% (875) |  |
| **Maternal education, % (n)** | |  |  | <0.001 |
|  | Up to upper secondary | 51.1% (3564) | 28.9% (2462) |  |
|  | Intermediate | 19.7% (1373) | 25.4% (2161) |  |
|  | 3-y university degree | 13.3% (926) | 21.8% (1859) |  |
|  | At least 5-y university degree | 15.9% (1108) | 23.8% (2029) |  |
| **Maternal migration history, % (n)** | |  |  | <0.001 |
|  | Immigrant | 14.8% (1132) | 6.1% (520) |  |
|  | Descendant of at least one immigrant | 12.5% (957) | 8.6% (733) |  |
|  | Rest of population | 72.6% (5544) | 85.3% (7258) |  |
| **Household income, % (n)** | |  |  | <0.001 |
|  | ≤1110 €/month | 34.9% (2369) | 14.2% (1208) |  |
|  | 1111-1500 €/month | 27.6% (1874) | 27.8% (2367) |  |
|  | 1501-1944 €/month | 18.8% (1276) | 28.1% (2388) |  |
|  | 1945-2500 €/month | 10.4% (706) | 19.3% (1641) |  |
|  | >2500 €/month | 8.2% (557) | 10.7% (907) |  |
| **Residence area, % (n)** | |  |  | <0.001 |
|  | Rural | 17.0% (1611) | 24.3% (2072) |  |
|  | Urban | 83.0% (7857) | 75.7% (6439) |  |
| **Mother smoking during pregnancy, % (n)** | |  |  | <0.001 |
|  | Never smoker | 55.6% (5101) | 58.8% (5003) |  |
|  | Smoker only before pregnancy | 19.3% (1770) | 26.4% (2249) |  |
|  | Smoker only in early pregnancy | 4.4% (405) | 3.5% (297) |  |
|  | Smoker throughout pregnancy | 20.7% (1906) | 11.3% (962) |  |
| **Mother weight status, % (n)** | |  |  | <0.001 |
|  | <18.5 kg/m² | 8.8% (804) | 6.8% (575) |  |
|  | 18.5–24.9 kg/m² | 62.1% (5653) | 67.4% (5733) |  |
|  | 25.0–29.9 kg/m² | 18.0% (1639) | 17.0% (1449) |  |
|  | ≥30.0 kg/m² | 11.1% (1013) | 8.8% (754) |  |
| **Parental stimulation ^a^, % (n)** | |  |  | <0.05 |
|  | Often | 64.9% (3428) | 67.8% (5771) |  |
|  | Sometimes | 33.0% (1740) | 30.5% (2596) |  |
|  | Rarely or never | 2.1% (110) | 1.7% (144) |  |
| **Child sex, % (n)** | |  |  | 0.30 |
|  | Boy | 51.8% (4887) | 51.1% (4346) |  |
|  | Girl | 48.2% (4547) | 48.9% (4165) |  |
| **Birth weight category ^b^, % (n)** | |  |  | <0.001 |
|  | Small for GA | 10.6% (948) | 8.9% (755) |  |
|  | Adequate for GA | 79.6% (7095) | 80.6% (6861) |  |
|  | Large for GA | 9.8% (873) | 10.5% (895) |  |
| **Gestational age in weeks, Mean (SD)** | | 39.1(1.5) | 39.3(1.4) | <0.001 |
| **Number of older siblings in the household, % (n)** | |  |  | <0.001 |
|  | First child | 43.0% (3343) | 45.8% (3901) |  |
|  | Second child | 34.9% (2708) | 38.0% (3232) |  |
|  | At least third child | 22.1% (1716) | 16.2% (1378) |  |
| **Collective care attendance, % (n)** | |  |  | <0.001 |
|  | First attendance by 4 months | 15.8% (822) | 25.2% (2149) |  |
|  | First attendance between 4 and 6 months | 7.9% (411) | 11.9% (1017) |  |
|  | First attendance between 6 and 12 months | 10.1% (528) | 12.4% (1059) |  |
|  | No collective care up to 12 months | 66.2% (3453) | 50.4% (4286) |  |
| Infant feeding practices | | | | |
| **Any breastfeeding duration in months, Mean (SD)** | |  |  | <0.001 |
|  | Never | 32.1% (2960) | 23.4% (1991) |  |
|  | Up to 1 month | 17.3% (1589) | 16% (1358) |  |
|  | Between 1 and 3 months | 10.4% (960) | 16.2% (1383) |  |
|  | Between 3 and 6 months | 22.7% (2089) | 19.2% (1638) |  |
|  | Between 6 and 9 months | 8.9% (818) | 12.2% (1038) |  |
|  | Beyond 9 months | 8.6% (794) | 13% (1103) |  |

## Part B: Adjustment variables

Confounding variables comprise sociodemographic and infant health variables (a full list is provided in Table S1). These were identified according to Direct Acyclic Graphs (DAGs) ^1^ as applied using Daggity v3.0 (Figure S1) ^2^.

### Definition of sociodemographic and infant health variables

Confounding variables are defined in Table S1. Adjusted models further considered three covariates relevant to the study design and recruitment, i.e. recruitment wave (four waves), maternity unit size (five strata based on yearly number of deliveries) and region of residence according to postal codes (nine regions in metropolitan France spreading North-South and West-East).

Table S2. Confounding variables: categorical variables (with their modalities) and continuous variables considered for multiple imputations (n=10089).

| **CONFOUNDING VARIABLES ^a^** | | |
| --- | --- | --- |
| **Variable** | **Modalities** | **Number of total missing data for each variable** |
| Socio-demographic and health | |  |
| **Maternal age at delivery** | <25 years  25-29 years  30-34 years  ≥35 years | 0 |
| **Maternal employment during pregnancy** | Employed  Unemployed  Out of the labour force (i.e., housewife, retired, students) | 6 |
| **Maternal education level** | Up to upper secondary  Intermediate  3-y university degree  At least 5-y university degree | 155 |
| **Maternal migration history** | Immigrant  Descendant of at least one immigrant  Rest of population | 26 |
| **Household income per consumption unit ^b^** | ≤1110 €/month  1111-1500 €/month  1501-1944 €/month  1945-2500 €/month  >2500 €/month | 264 |
| **Residence area size ^c^** | Rural  Urban | 1 |
| **Mother smoking during pregnancy** | Never smoker  Smoker only before pregnancy  Smoker only in early pregnancy  Smoker throughout pregnancy | 115 |
| **Mother BMI status** | <18.5 kg/m²  18.5–24.9 kg/m²  25.0–29.9 kg/m²  ≥30.0 kg/m² | 126 |
| **Parental stimulation ^d^** | Often  Sometimes  Rarely or never | 288 |
| **Child sex** | Boy  Girl | 0 |
| **Birth weight category ^e^** | Small for GA  Adequate for GA  Large for GA | 282 |
| **Gestational age in weeks** | - | 152 |
| **Number of older siblings in the household** | First child  Second child  At least third child | 2 |
| **Collective care attendance** | First attendance by 4 months  First attendance between 4 and 6 months  First attendance between 6 and 12 months  No collective care up to 12 months | 519 |
| Infant feeding practices | |  |
| **Any breastfeeding duration in months** | Never  Up to 1 month  Between 1 and 3 months  Between 3 and 6 months  Between 6 and 9 months  Beyond 9 months | 96 |
| **Age at introduction to complementary feeding** | Earlier than 4 months  Between 4 and 6 months  After 6 months | 224 |
| ^a^ A full description of data collection and management in the ELFE cohort can be found in the publication outlining the cohort profile ^3^ and in publications focusing on feeding practices ^4,5^  ^b^ Estimation of the household income per consumption unit according to the definition by INSEE (French national institute for statistical and economic studies) ^6^  ^c^ Identification of urban/rural areas based on postal codes  ^d^ Parental stimulation was defined according to the frequency of activities (e.g., drawing, playing) with the child, as reported by mothers at the 1-year follow-up ^3,7^  ^e^ Birth weight for gestational age was evaluated according to the Audipog reference curves ^8^ | | |

### Direct Acyclic Graphs

Below is shown the figure corresponding to the code on Daggity v 3.0, which identified relevant confounding variables (Figure S1).


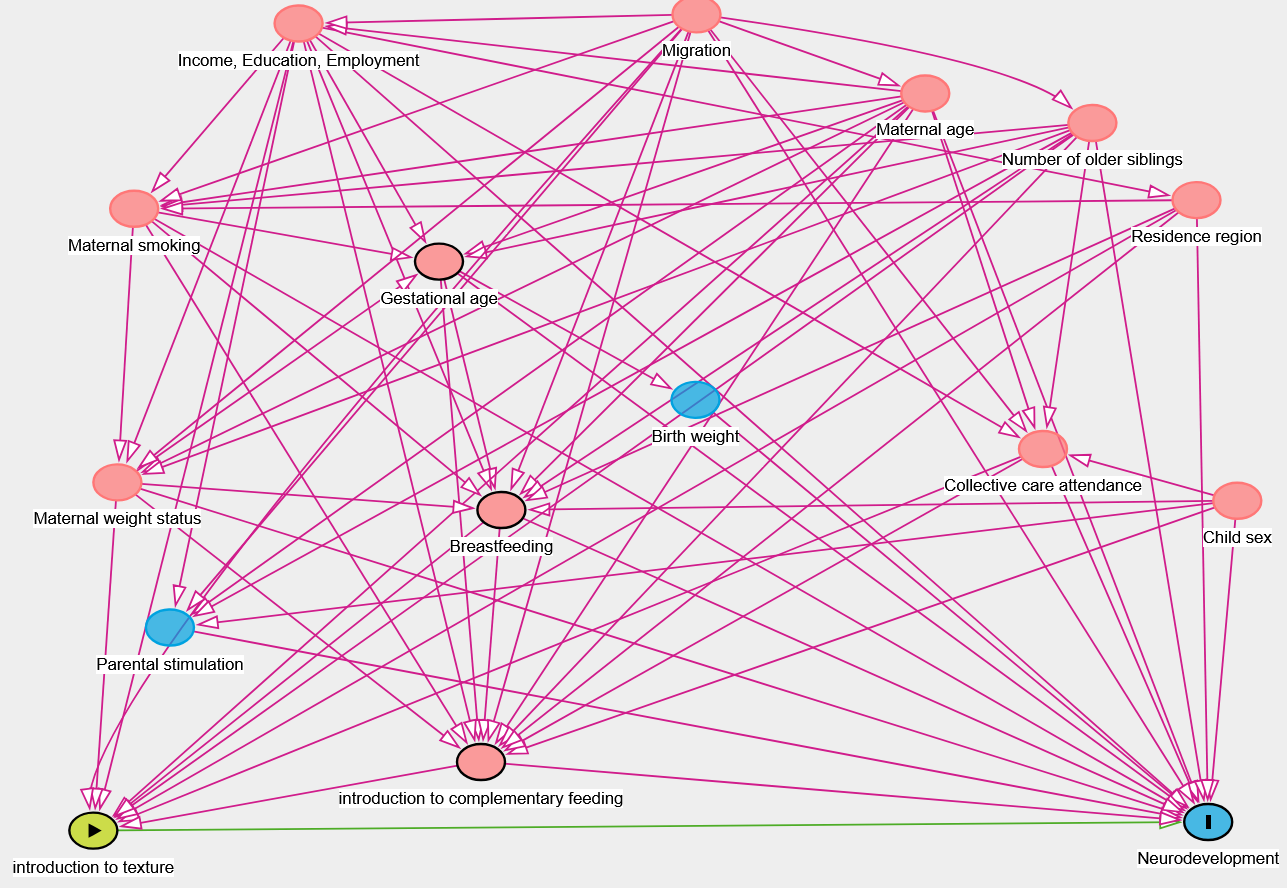


Figure S1. Direct Acyclic Graph (DAGs) applied to adjusted analyses throughout the manuscript using Daggity v 3.0^2^. Green circle: the main exposure (age at introduction to food texture). Blue circle: the outcome (neurodevelopmental scores). Potential confounders are depicted by red circles, whilst variables that are not expected to influence exposure but are related to the outcome are depicted by blue circles. Arrows denote the direction of causal association. The green arrow illustrates the direct associations between exposure and outcome in the present paper.

The thick blue arrow highlights the direction of associations between feeding practices, i.e. introduction to complementary feeding, towards introduction to texture.

## Part C: Supplementary Tables showing fitted models

Table S3. Adjusted estimates (95% CI) and ORs (95% CI) of neurodevelopmental outcomes in total sample, weighted and imputed analyses.

|  |  | **Neurodevelopmental scores (standardised continuous)** | | | | **At risk for developmental delay (binary)** |
| --- | --- | --- | --- | --- | --- | --- |
|  |  | **CDI-1** | **MB-2** | **CDI-3.5** | **PS-3.5** | **DQ-3.5<90** |
|  |  | **Estimate [95% CI]** | **Estimate [95% CI]** | **Estimate [95% CI]** | **Estimate [95% CI]** | **OR [95% CI]** |
| *Total sample, weighted ^a^* | | *N=8510* | *N=7948* | *Ν=7579* | *Ν=6207* | *N=7579* |
|  | Between 8 and 10 months | **-0.15 [-0.21; -0.08]** | **-0.12 [-0.20; -0.05]** | **-0.13 [-0.21; -0.04]** | -0.03 [-0.12; 0.05] | 1.20 [0.93; 1.54] |
|  | After 10 months | **-0.39 [-0.46; -0.31]** | **-0.20 [-0.28; -0.12]** | **-0.21 [-0.30; -0.11]** | -0.03 [-0.12; 0.07] | **1.70 [1.31; 2.20]** |
| *Total sample, imputed ^a^* | | *N=9869* | *N=9309* | *Ν=8854* | *Ν=7200* | *Ν=8854* |
|  | Between 8 and 10 months | **-0.13 [-0.17; -0.09]** | **-0.06 [-0.10; -0.01]** | -**0.06 [-0.11; -0.02]** | -0.02 [-0.07; 0.04] | 1.09 [0.93; 1.28] |
|  | After 10 months | **-0.37 [-0.42; -0.32]** | **-0.13 [-0.18; -0.08]** | **-0.17 [-0.22; -0.12]** | -0.04 [-0.10; 0.02] | **1.57 [1.33; 1.85]** |
| CDI-1: Child Development Inventory at 1 year; MB-2: McArthur-Bates Communicative Development Inventory at 2 years; CDI-3.5: Child Development Inventory at 3.5 years; PS-3.5: Pictures Similarities subscale of the British Ability Scale at 3.5 years; DQ-3.5<90: Developmental Quotient (developmental age/chronological age x 100) below 90 indicating a possible risk for developmental delay.  Estimates (95% CI) for CDI-1, MB-2, CDI-3.5 and PS-3.5 were calculated using linear regression models. Reference category [0] is introduction to food pieces before 8 months.  ORs (95% CI) for DQ-3.5<90 were calculated using binary logistic regression models. Reference category [1] is introduction to food pieces before 8 months.  ^a^ Adjustments considered the following confounding variables and covariates: maternal age at delivery, maternal employment, maternal smoking during pregnancy, maternal education level, maternal migration history, household income per consumption unit, residence area size, maternal BMI status before pregnancy, parental stimulation, child sex, child birth weight category, child gestational age, number of older siblings in the household, collective care attendance, breastfeeding duration and age of introduction to complementary feeding, along with recruitment wave, maternity unit and region of residence. Age at neurodevelopmental assessment was further considered, when applicable, i.e. in the linear regression models for CDI-1, MB-2, CDI-3.5 and PS-3.5. | | | | | | |

Table S4. Adjusted estimates (95% CI) and ORs (95% CI) of neurodevelopmental outcomes in separate models for introduction to food pieces in terms of fruits/vegetables and meat, respectively, complete-case analyses.

|  |  | **CDI-1** | **MB-2** | **CDI-3.5** | **PS-3.5** | **DQ-3.5<90** |
| --- | --- | --- | --- | --- | --- | --- |
|  |  | **Estimate [95% CI]** | **Estimate [95% CI]** | **Estimate [95% CI]** | **Estimate [95% CI]** | **OR [95% CI]** |
| Age at introduction to food pieces (fruits/vegetables) | | | | | | |
| *Total sample ^a^* | | *N=8493* | *N=7932* | *Ν=7564* | *Ν=6192* | *N=7564* |
|  | Between 8 and 10 months | **-0.12 [-0.16; -0.07]** | **-0.06 [-0.11; -0.01]** | **-0.07 [-0.12; -0.02]** | -0.02 [-0.08; 0.03] | 1.11 [0.93; 1.33] |
|  | After 10 months | **-0.35 [-0.40; -0.30]** | **-0.15 [-0.20; -0.01]** | **-0.17 [-0.22; -0.12]** | -0.04 [-0.10; 0.02] | **1.56 [1.31; 1.87]** |
| Age at introduction to food pieces (meat) | | | | | | |
| *Total sample ^a^* | | *N=7548* | *N=7105* | *Ν=6791* | *Ν=5587* | *Ν=6791* |
|  | After 10 months | **-0.24 [-0.29; -0.20]** | **-0.09 [-0.13; -0.04]** | **-0.14 [-0.19; -0.10]** | 0 [-0.06; 0.05] | **1.37 [1.16; 1.62]** |
| CDI-1: Child Development Inventory at 1 year; MB-2: McArthur-Bates Communicative Development Inventory at 2 years; CDI-3.5: Child Development Inventory at 3.5 years; PS-3.5: Pictures Similarities subscale of the British Ability Scale at 3.5 years; DQ-3.5<90: Developmental Quotient (developmental age/chronological age x 100) below 90 indicating a possible risk for developmental delay.  Estimates (95% CI) for CDI-1, MB-2, CDI-3.5 and PS-3.5 were calculated using linear regression models. Reference category [0] is introduction to food pieces before 8 months.  ORs (95% CI) for DQ-3.5<90 were calculated using binary logistic regression models. Reference category [1] is introduction to food pieces before 8 months.  ^a^ Adjustments considered the following confounding variables and covariates: maternal age at delivery, maternal employment, maternal smoking during pregnancy, maternal education level, maternal migration history, household income per consumption unit, residence area size, maternal BMI status before pregnancy, parental stimulation, child sex, child birth weight category, child gestational age, number of older siblings in the household, collective care attendance, breastfeeding duration and age of introduction to complementary feeding, along with recruitment wave, maternity unit and region of residence. Age at neurodevelopmental assessment was further considered, when applicable, i.e. in the linear regression models for CDI-1, MB-2, CDI-3.5 and PS-3.5. | | | | | | |

Table S5. Adjusted ORs of having a poor developmental sub-score across categories of increasing age at introduction to food pieces, complete-case analyses at 1-year follow-up (n=8511) and at 3.5 years follow-up (n=7580).

| Poor developmental sub-score (lowest quartile v. three other quartiles) ^a^ | | | | | | | |
| --- | --- | --- | --- | --- | --- | --- | --- |
|  |  | Social skills | Self-help | Gross motor skills | Fine motor skills | Language expression | Language comprehension |
|  |  |  |  |  |  |  |  |
| 1-year follow-up  % (n) ^a^ | | 19.2% (1631) | 10.1% (863) | 19% (1618) | 25.2% (2147) | 17% (1450) | 17.6% (1495) |
| Estimates ^b^ | | OR (95% CI) | OR (95% CI) | OR (95% CI) | OR (95% CI) | OR (95% CI) | OR (95% CI) |
|  | Between 8 and 10 months | 1.10 [0.96 ; 1.26] | 1.19 [0.99 ; 1.43] | **1.35 [1.17 ; 1.55]** | 1.02 [0.90 ; 1.15] | **1.34 [1.16 ; 1.54]** | 1.14 [0.99 ; 1.31] |
|  | After 10 months | **1.5 [1.30 ; 1.73]** | **2.16 [1.80 ; 2.59]** | **1.70 [1.47 ; 1.96]** | **1.44 [1.27 ; 1.64]** | **1.77 [1.52 ; 2.06]** | **1.45 [1.25 ; 1.68]** |
| 3-year follow-up  % (n) ^a^ | | 14.5% (1101) | 12.8% (969) | 10.6% (803) | 19.9% (1506) | 16.3% (1234) | 14.9% (1133) |
| Estimates ^b^ | | OR (95% CI) | OR (95% CI) | OR (95% CI) | OR (95% CI) | OR (95% CI) | OR (95% CI) |
|  | Between 8 and 10 months | 1.13 [0.97 ; 1.33] | 0.99 [0.83 ; 1.17] | 1.08 [0.90 ; 1.30] | 1.06 [0.92 ; 1.22] | **1.23 [1.05 ; 1.43]** | **1.21 [1.03 ; 1.42]** |
|  | After 10 months | 1.07 [0.90 ; 1.27] | **1.31 [1.10 ; 1.56]** | 1.21 [1.00 ; 1.46] | **1.32 [1.13 ; 1.54]** | **1.50 [1.27 ; 1.77]** | **1.36 [1.15 ; 1.61]** |

^a^ Poor developmental score: <25^th^ percentile, social skills: At 1 year <6 and at 3·5 years <9, self-help: At 1 year <4 and at 3·5 years <7, gross motor skills: At 1 year <3 and at 3·5 years <8, fine motor skills: At 1 year <7 and at 3.5 years <6, language expression: At 1 year <5 and at 3·5 years <9, language comprehension: At 1 year <7 and at 3·5 years <8.

^b^ Adjustments considered the following confounding variables and covariates: maternal age at delivery, maternal employment, maternal smoking during pregnancy, maternal education level, maternal migration history, household income per consumption unit, residence area size, maternal BMI status before pregnancy, parental stimulation, child sex, child birth weight category, child gestational age, number of older siblings in the household, collective care attendance, breastfeeding duration and age of introduction to complementary feeding, along with recruitment wave, maternity unit and region of residence. Age at neurodevelopmental assessment was further considered. ORs (95% CI) were calculated using binary logistic regression models. Reference category [1] is introduction to food pieces before 8 months.

1. Ferguson KD, McCann M, Katikireddi SV, et al. Evidence synthesis for constructing directed acyclic graphs (ESC-DAGs): a novel and systematic method for building directed acyclic graphs. *Int J Epidemiol.* 2020;49(1):322-329.

2. Textor J, van der Zander B, Gilthorpe MS, Liskiewicz M, Ellison GT. Robust causal inference using directed acyclic graphs: the R package 'dagitty'. *Int J Epidemiol.* 2016;45(6):1887-1894.

3. Charles MA, Thierry X, Lanoe JL, et al. Cohort Profile: The French national cohort of children (ELFE): birth to 5 years. *Int J Epidemiol.* 2020;49(2):368-+.

4. Wagner S, Kersuzan C, Gojard S, et al. Breastfeeding initiation and duration in France: The importance of intergenerational and previous maternal breastfeeding experiences - results from the nationwide ELFE study. *Midwifery.* 2019;69:67-75.

5. Bournez M, Ksiazek E, Wagner S, et al. Factors associated with the introduction of complementary feeding in the French ELFE cohort study. *Maternal & child nutrition.* 2018;14(2):e12536.

6. INSEE. *Définitions, Méthodes et Qualité* 2022; <https://www.insee.fr/fr/metadonnees/definition/c1802>. Accessed 31 January 2023.

7. Martinot P, Adjibade M, Taine M, et al. LC-PUFA enrichment in infant formula and neurodevelopment up to age 3.5 years in the French nationwide ELFE birth cohort. *Eur J Nutr.* 2022;61(6):2979-2991.

8. Mamelle N, Munoz F, Grandjean H. *J Gynécologie Obstétrique Biol Reprod.* 1996;25(1):61-70.
